# A randomized, 12-month controlled trial to evaluate non-inferiority of early compared to conventional loading of modSLA implants in single tooth gaps

**DOI:** 10.1186/s40729-016-0040-8

**Published:** 2016-04-04

**Authors:** Michel Dard, Makoto Shiota, Minoru Sanda, Yasutomo Yajima, Hideshi Sekine, Shohei Kasugai

**Affiliations:** 1 College of Dentistry, New York University, New York, NY USA; 2 Tokyo Medical and Dental University, Tokyo, Japan; 3 Suidobashi Hospital, Tokyo Dental College, Tokyo, Japan; 4 School of Dentistry, Ohu University, Fukushima, Japan; 5 Department of Oral Implantology and Regenerative Dental Medicine, Tokyo Medical and Dental University, 1-5-45 Yushima, Bunkyo-ku, Tokyo, 113-8549 Japan

**Keywords:** Early loading, Conventional loading, Non-inferiority, Crestal bone level, Maxilla, Mandible

## Abstract

**Background:**

The aim of the study was to evaluate whether early loading of implants with a chemically modified sandblasted, large-grit, acid-etched (SLA) (SLActive®) surface was non-inferior to conventional loading in terms of change in crestal bone level.

**Methods:**

This was a randomized, controlled, multicenter study. Patients requiring single-tooth rehabilitation in the posterior maxilla or mandible received implants and were randomized to receive a provisional restoration in occlusal load after 25 ± 3 days (early loading) or after 13 ± 1 weeks (conventional loading). The primary endpoint was change in crestal bone level between implant placement (baseline) and 6 months. Secondary endpoints included change in crestal bone level between baseline and 12 months, implant survival and success rates, and patient satisfaction.

**Results:**

Of the 84 patients enrolled, 78 received implants and were randomized onto the early loading (41 patients) and conventional loading (37 patients) groups. The mean change in crestal bone level between baseline and 6 months was 0.56 ± 0.58 and 0.51 ± 0.62 mm for early and conventional loading, respectively; at 12 months, the mean change was 0.76 ± 0.60 and 0.73 ± 0.77 mm, respectively. Implant survival and success at 12 months were 100 % for both groups. Patient satisfaction was similar between the groups, except that more patients in the early loading group were satisfied or highly satisfied with the time taken for fitting.

**Conclusion:**

The study demonstrated that early implant loading was non-inferior to conventional implant loading in terms of crestal bone level change in a Japanese patient population in short follow-up period and single tooth gaps in molar regions.

## Background

The use of dental implants to replace missing or compromised teeth has been well documented clinically over many years. High implant survival rates have been demonstrated for over 10 [1–3], 15 [4], and 20 years [5, 6]. Long-term survival rates for single-tooth implants have been shown to be greater than those for tooth-supported restorations, e.g., fixed partial dentures (FPDs) [7, 8]. Good long-term survival rates have also been observed in the posterior regions of the jaws, i.e., in premolar and molar regions [1, 9, 10].

The conventional protocol for loading of rough-surfaced dental implants, i.e., placement of the prosthetic crown, recommends undisturbed healing after surgery for 3 months in the mandible and 4–6 months in the maxilla [11, 12] to allow the osseointegration process to take place. However, it would be beneficial if the healing period could be shortened without jeopardizing implant success [13]. Earlier implant loading protocols, e.g., loading after 3–4 weeks, have therefore been investigated. Such protocols may also have advantages in terms of preservation of the soft and hard tissues [14]. Early loading has also shown equivalence to conventional loading in terms of the amount of marginal peri-implant bone loss [12, 15] or implant or prosthesis failure [12]. Similar outcomes have also been demonstrated between early and conventional loading in the first molar region in the maxilla and mandible [16].

Early loading still requires osseointegration to take place. Implant surfaces have therefore been developed to try to speed up the osseointegration process. Some of these implant surface modifications have included alteration of the surface features to produce micro-rough, micro-porous, or nano-rough surface topography [17–19] or biochemical modification through impregnation, coating, or processing [20–22].

One of the more clinically successful implant surface modifications in recent years was the chemical modification of the sandblasted, large-grit, acid-etched (SLA) surface to produce the hydrophilic SLActive® surface (Straumann AG, Basel, Switzerland). It is accomplished by rinsing the titanium surface after the etching process under nitrogen protection and continuous storage in an isotonic NaCl solution that prevent deposition of carbon compounds. Preclinical data have demonstrated significantly greater bone-to-implant contact [23–25], bone fill [26], and removal torque [27] with the modified SLA surface compared to conventional SLA. Earlier osseointegration has also been demonstrated histologically [28–30]. Clinical results have shown greater implant stability [31] and an earlier shift from decreasing to increasing implant stability after placement [32]. The original SLA surface allowed the implant restoration time to be reduced from 12 to 6 weeks [33, 34]; the properties of the chemically modified SLA surface allow this time to be further reduced to 3–4 weeks. Early loading with these implants has been demonstrated in a number of clinical trials [35–38].

**Fig. 1:**
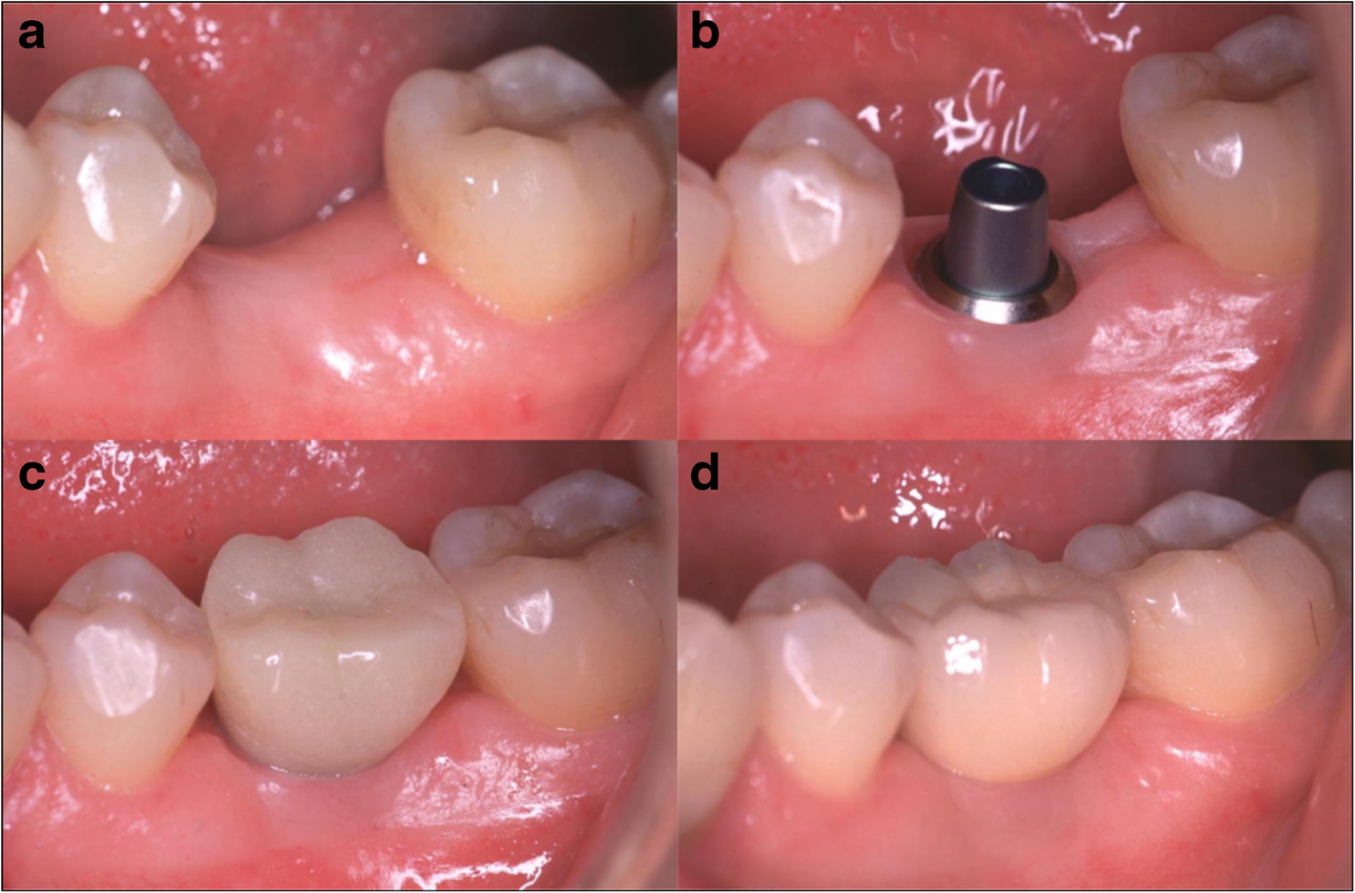
Clinical pictures in each procedure. **a** Before implant placement. **b** After abutment connection. **c** Temporary prosthesis. **d** Final prosthesis

Early loading protocols have become relatively common procedures in many countries for implant restoration, but the procedure is much less common in Japan. The purpose of this study, therefore, was to investigate whether the chemically modified SLA implant with early loading was non-inferior to conventional loading, based on the amount of crestal bone change between baseline and 6 months after surgery (Fig. 1).

## Methods

This study was designed as a randomized, controlled, multicenter clinical trial to evaluate non-inferiority of early loading compared to conventional loading of dental implants with a chemically modified SLA surface placed in single tooth gaps, involving three centers in Japan (Tokyo Medical and Dental University (TMDU), Tokyo Dental College Chiba Hospital (TDCC), and Tokyo Dental College Suidobashi Hospital (TDCS)). The study was conducted in accordance with the Declaration of Helsinki (1964 and all subsequent amendments), the Japanese Pharmaceutical Affairs Law, the Ordinance Concerning the Standards for Clinical Trials on Medical Devices (2005 MHLW Ordinance No. 36), and the relevant notifications and protocol. The study was approved by the Institutional Review Boards of the medical institutions involved. Written informed consent was obtained from all patients. The study was registered at www.clinicaltrials.gov.

### Patients

Patients were enrolled according to strict pre-defined inclusion and exclusion criteria. The most important inclusion criteria were as follows: age ≥20 years, single tooth gaps in molar or premolar in the mandible or maxilla, bone quality I–III and sufficient bone quantity to allow implant placement, and substantially healed extraction sockets (at least 16 weeks after tooth extraction).

Important exclusion criteria fell into two categories: systemic and dental. The most important systemic exclusion criteria were as follows: systemic disease, e.g., diabetes mellitus; serious internal medical problems, e.g., cardiac or cerebral infarction; bone disorders, e.g., metabolic bone disease, temporomandibular joint disorders, treatable changes in the oral mucosa, local root remnants, xerostomia, and bisphosphonate medication; inadequate wound healing capacity, prolonged therapy-resistant functional disorders, and steroid use or irradiation; uncontrolled bleeding disorders and anticoagulation drugs/hemorrhagic diatheses; psychoses, drug or alcohol abuse, or titanium allergy (based on patient declaration); smoking >10 cigarettes per day; pregnancy and/or breastfeeding; participation in another clinical trial during or within 30 days before this trial; unwillingness or inability to follow the investigator’s instructions; or any other conditions that might prevent study completion in the opinion of the investigator. The dental exclusion criteria were as follows: untreated dental and serious periodontal lesions; severe bruxism or clenching habits; existing implants in the adjacent position; removable dentures or un-restored tooth gaps in the opposing dentition; probing pocket depth ≥4 mm at a tooth immediately adjacent to the dental implant site; major simultaneous augmentation procedures; requirement for maxillary sinus lift, socket preservation, or ridge augmentation; and failure of a previous implant at the planned implant site (Table 1).

**Table 1:** Inclusion criteria and exclusion criteria

Inclusion criteria	Age over 20 Patient who have missing teeth in premolar or molar site Good oral hygiene Predicted implant site has 1–3 quality of bone densityand enough quantity of bone Extraction socket in predicted implant site is completely healed (16 weeks or more)
Exclusion criteria	Systemic condition negatively affect implant treatment (e.g., septicemia, immune deficiency, diabetes) Systemic condition that contraindicate oral surgical procedure Patient with ten or more cigarette consumption in a day Patient who is going to participate in another clinical trial or already joined within 30 days before agreement of this trial Patient who do not follow and cooperative dentist’s instruction Pregnant or lactating female or female who might have willingness to be pregnant Patient who has caries or severe periodontal disease Severe parafunction of bruxism or clenching Antagonist of expected implant site is removable partial denture or edentulous site without prosthesis Patient with poor oral hygiene or not positive for plaque control Patient have adjacent teeth next to the edentulous site with periodontal pocket of 4 mm or more Cases needs bone augmentation procedure History of implant failure at the same site

When a patient fulfilled all inclusion criteria and had no exclusion criteria, then he/she got implant surgery and checked the condition met first criteria for loading (loading criteria 1 (LC1)) (Table 2).

**Table 2:** Loading criteria applied at implant placement surgery (loading criteria 1 (LC1)) and attachment of provisional restoration (loading criteria 2 (LC2))

Loading criteria 1	Loading criteria 2
• Sufficient oral hygiene • At least 1 mm bone volume around the implant^a^ • No major dehiscence (<3 mm) or other bone defects at the implant site • Bone quality I–III • Adequate insertion torque (≥15 Ncm during placement of the healing cap) • Suitable implant position • Dental radiograph shows three threads of implant fixture	• Sufficient oral hygiene • No rotational movement of the implant^b^ during abutment connection at 15 Ncm • No moderate or severe pain at the implant site during abutment connection at 15 Ncm • Suitable implant position • Dental radiograph shows three threads of implant fixture

Any patients not fulfilling LC1 received an alternative treatment, e.g., bridge, false teeth, and were subsequently included in the safety analysis set (SAS)

^a^That is, for a 4.1-mm diameter 10-mm-long implant, crestal width and bone height should be 6.1 and 11 mm, respectively

^b^During abutment connection

### Implants

All patients received Ti grade IV Straumann Standard Plus Regular Neck (SP RN) implants, 4.1 mm in diameter and 8, 10, or 12 mm in length, with SLActive® surface (Institut Straumann AG, Basel, Switzerland).

### Randomization

Patients fulfilling the necessary criteria were randomized to the early loading arm (implant loading after 25 ± 3 days) or conventional loading arm (implant loading after 13 ± 1 weeks). The initial randomization sequence was created after implant surgery once the previously defined LC1 had been met, by means of variable block sizes in order to avoid disproportionate allocation within early loading or conventional loading groups [39]. Lists were prepared by an independent statistician and were centrally controlled by a third party who was not involved in the study. All patients who fulfilled LC1 were included in the full analysis set (FAS). If patients did not fulfill LC1, they received an alternative treatment, e.g., bridge, false teeth, or treatment by other implant, and were subsequently included and evaluated in the safety analysis set (SAS).

**Fig. 2:**
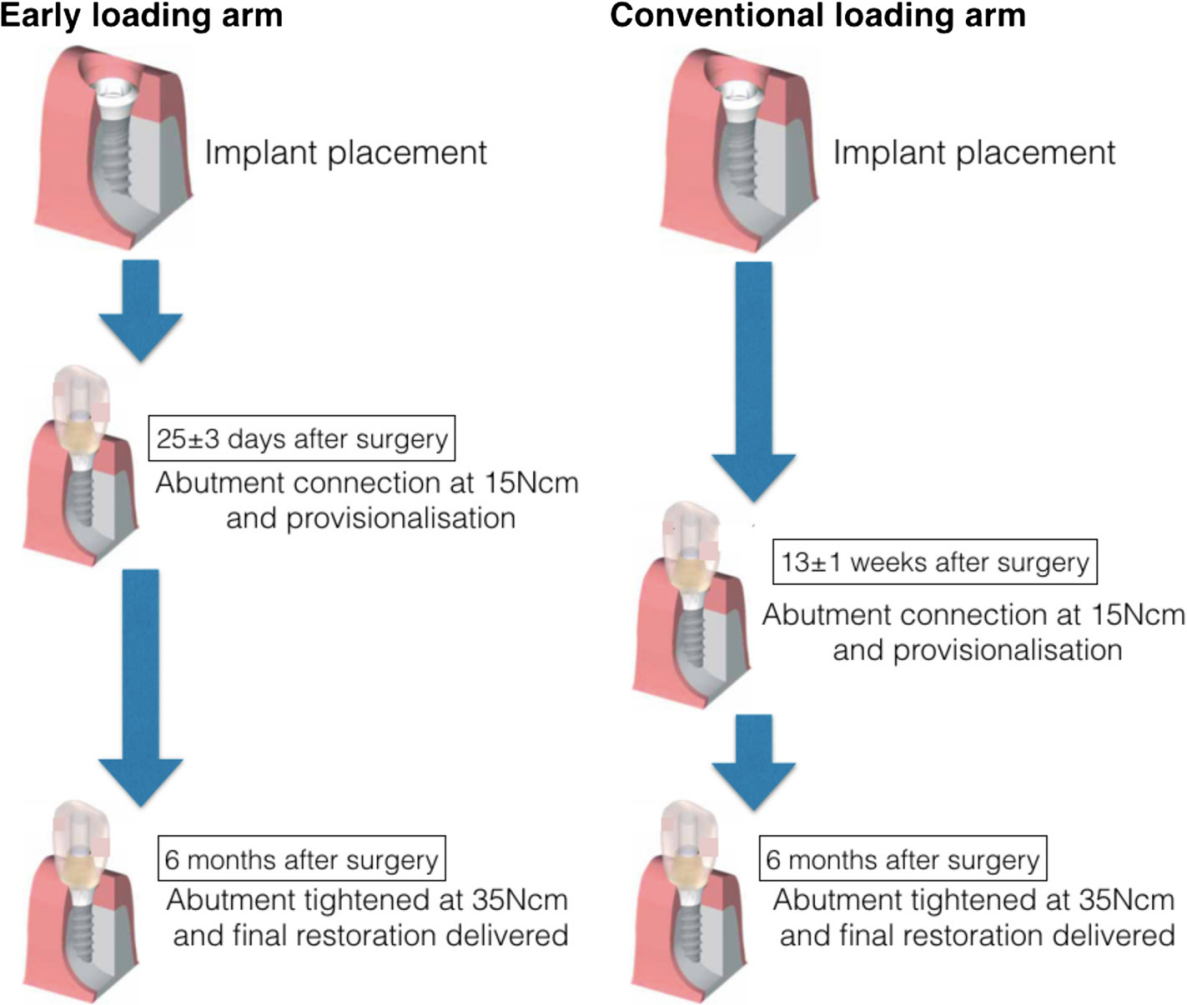
Restorative flow diagram

Loading criteria were evaluated once more (loading criteria 2 (LC2); Table 2) at the time of provisional restoration delivery at 25 ± 3 days after surgery in the early loading arm and at 13 ± 1 weeks after surgery in the conventional loading arm (Fig. 2). Patients fulfilling the LC2 criteria were included in the per protocol set (PPS). If patients did not fulfill LC2 (i.e., due to an unstable implant or moderate to severe pain), they received an alternative treatment and were included in the FAS. All patients receiving an implant were therefore included in the FAS irrespective of whether they fulfilled LC2.

### Surgical and restoration procedure

There were six evaluation time points from recruitment to study completion with a variety of safety and efficacy data obtained at each time point, including primary and secondary endpoint data. The day of implant surgery (day 0) was the baseline time point; patient consent and screening procedures were performed between 8 weeks and 1 day before day 0. Implant surgery and placement were performed according to the manufacturer’s recommended guidelines. All implants were placed in the alveolar ridge that had healed for at least 16 weeks after tooth extraction. Sutures were removed 7–14 days after surgery. In both arms, ready-made abutments for cement retention were connected at 15 Ncm and implants were loaded through temporary crown. The occlusal contacts were equivalated as holding a 21-μm AccuFilm II (Parkell Inc, Edgewood, NY, USA) when patients bite heavily. Final crowns were placed 6 months after implant placement in both groups, with the same abutment for the temporary crown, and it was tightened at the torque of 35 Ncm. Patients were recalled for a follow-up evaluation 12 months after surgery.

### Efficacy evaluations

The primary endpoint was a change of crestal bone level between implant surgery (baseline) and final restoration (6 months), assessed by measuring the distance from the implant shoulder to the first bone-to-implant contact both mesially and distally to the implant.

Bone level was measured by a single reader on standardized periapical radiographs taken at baseline (day 0), suture removal (7–14 days after surgery), provisional restoration, final restoration, and at the 12-month follow-up. Radiographs were standardized by using customized film holders. A commercially available film holder was employed (e.g., System (Dentsply Rinn, Elgin, IL, USA)), RWT® window x-ray system (Kentzler-Kaschner Dental, Ellwangen, Germany) or similar). The film was placed almost parallel to the implants. Indentations of the incisal edge of the implant and of the neighboring teeth (where possible) were taken with impression material to improve reproducibility. The customized radiographic holder was fabricated by putting autopolymerizing resin to the biting plate of the film holder and adapted its shape to the patient dentition in order to standardize the position of the X-ray film. The radiograph was exposed once the resin had polymerized, and the stent removed and stored for future use.

Secondary endpoints included implant survival and success rates, changes in crestal bone level between baseline and 12 months, and patient satisfaction. Implant success and survival were assessed at suture removal, provisional restoration, final restoration, and at the 12-month follow-up. Implant survival was defined as remaining of implant, and implant success was defined according the criteria by Buser et al. [40], i.e., absence of pain, foreign body discomfort or dysesthesia, absence of recurrent peri-implant infection with suppuration, absence of implant mobility, and absence of continuous peri-implant radiolucency.

Patient satisfaction was evaluated at the final restoration and 12-month follow-up visits by asking the patients to rate their assessment of six parameters: prosthesis comfort, appearance, ability to chew, ability to taste, general satisfaction, and patients substantial feeling for adaptation. Patients rated their assessment on a five-point scale (highly satisfied, satisfied, no opinion, dissatisfied, highly dissatisfied).

In addition, periodontal examination, in the form of probing depth (PD) and bleeding on probing (BoP), was performed at pre-screening and at the 12-month follow-up.

### Statistical analysis

Descriptive summary statistics were computed for all parameters, and quantitative parameters were described using mean, standard deviation, median, quartiles, minimum, and maximum. For qualitative variables, absolute and relative frequencies were given. All descriptions were done separately for treatment groups and visits.

The hypothesis was that the change in crestal bone level between baseline and 6 months would be non-inferior for early loading compared to conventional loading. Non-inferiority was defined as a clinically relevant difference of up to 0.3 mm because in the same type of study by Bornstein et al. [35], most implants demonstrated 0.0 to 0.3 mm after 3 years of observation period, and it did not reach statistical significance. The null hypothesis (H0) was therefore that the mean crestal bone loss is >0.3 mm higher with early loading compared to conventional loading. The hypothesis was tested by calculating whether the difference of crestal bone loss was within 0.3 mm between conventional loading and early loading at 6 months (*p* < 0.05).

### Analysis data set

There were three types of data sets used: PPS, FAS, and SAS. The SAS included all patients who got implant treatment, including patients who did not meet LC1 and evacuated before randomization. The FAS included all patients who received an implant and who had at least one post-randomization measurement, irrespective of any premature termination or major protocol violations; this set therefore includes the PPS. The PPS includes all patients who completed the study with no major protocol violations.

Based on a two-group one-sided *t* test with significance level of 0.025, a sample size of 29 patients per group was calculated to have 80 % power to reject the null hypothesis, assuming an expected difference in means of 0, a common standard deviation of 0.4, and a non-inferiority lower limit of 0.3 mm. A subject drop-out rate of 20 % was assumed, giving a sample size of 37 patients per group (total of 74 patients). Calculations were made using nQuery Advisor 6.01.

## Results

### Patients

**Fig. 3:**
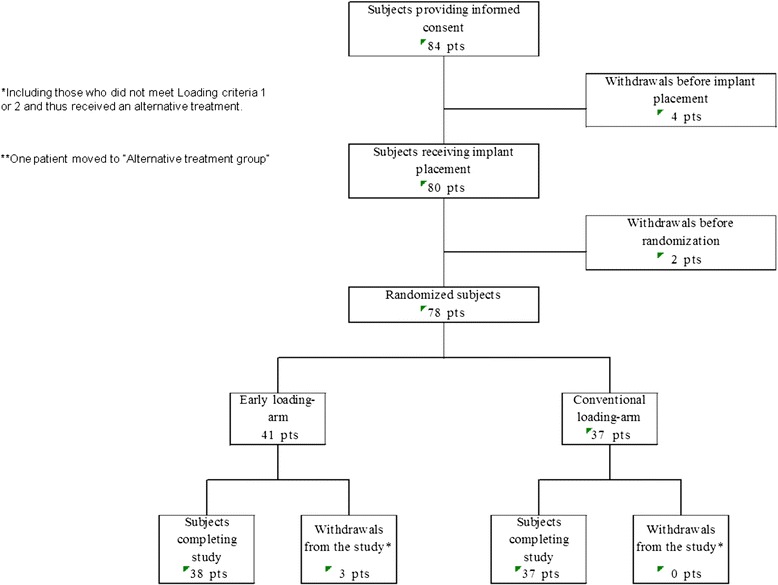
Participant flow diagram

The study enrolled 84 Japanese patients who had single missing tooth in the molar region. Since four patients were withdrawn due to the exclusion criteria (systemic disease, adjacent teeth with probing pocket depth deeper than 4 mm, mental disorder, and bone deficiency, respectively), 80 patients underwent implant placement. Two further patients were withdrawn before randomization because the insertion torque did not reach the 15 Ncm in the surgery which was one of the requirements for LC1. Of the 78 who participated in the study, 41 were allocated to the early loading arm. Three patients were withdrawn because implants in two patients lost their osseointegration to the alveolar bone and the other one an X-ray picture did not include three threads that had to be included for evaluation. Thirty seven patients were allocated to the conventional loading arm. The pregnancy was detected to one patient in the conventional loading arm, and she was excluded from the PPS and FAS. The full participant flow diagram is shown in Fig. 3.

The mean age at implant surgery in the PPS was 47.0 ± 14.5 years. Patient demographic data are shown in Table 3. The first patients were recruited in December 2010, and the final examination was performed in August 2012. No methodological changes were made after commencement of the trial.

**Table 3:** Patient demographics and other baseline characteristics (PPS)

Characteristics		Early loading arm	Conventional loading arm	Total
		(*N* = 38)	(*N* = 37)	(*N* = 75)
Age	No. of patients	38	37	75
	Mean	46.6	47.4	47.0
	SD	13.2	16.0	14.5
Gender	Male	5 (13.2)	19 (51.4)	24 (32.0)
	Female	33 (86.8)	18 (48.6)	51 (68.0)

### Efficacy evaluations: bone level changes

**Fig. 4:**
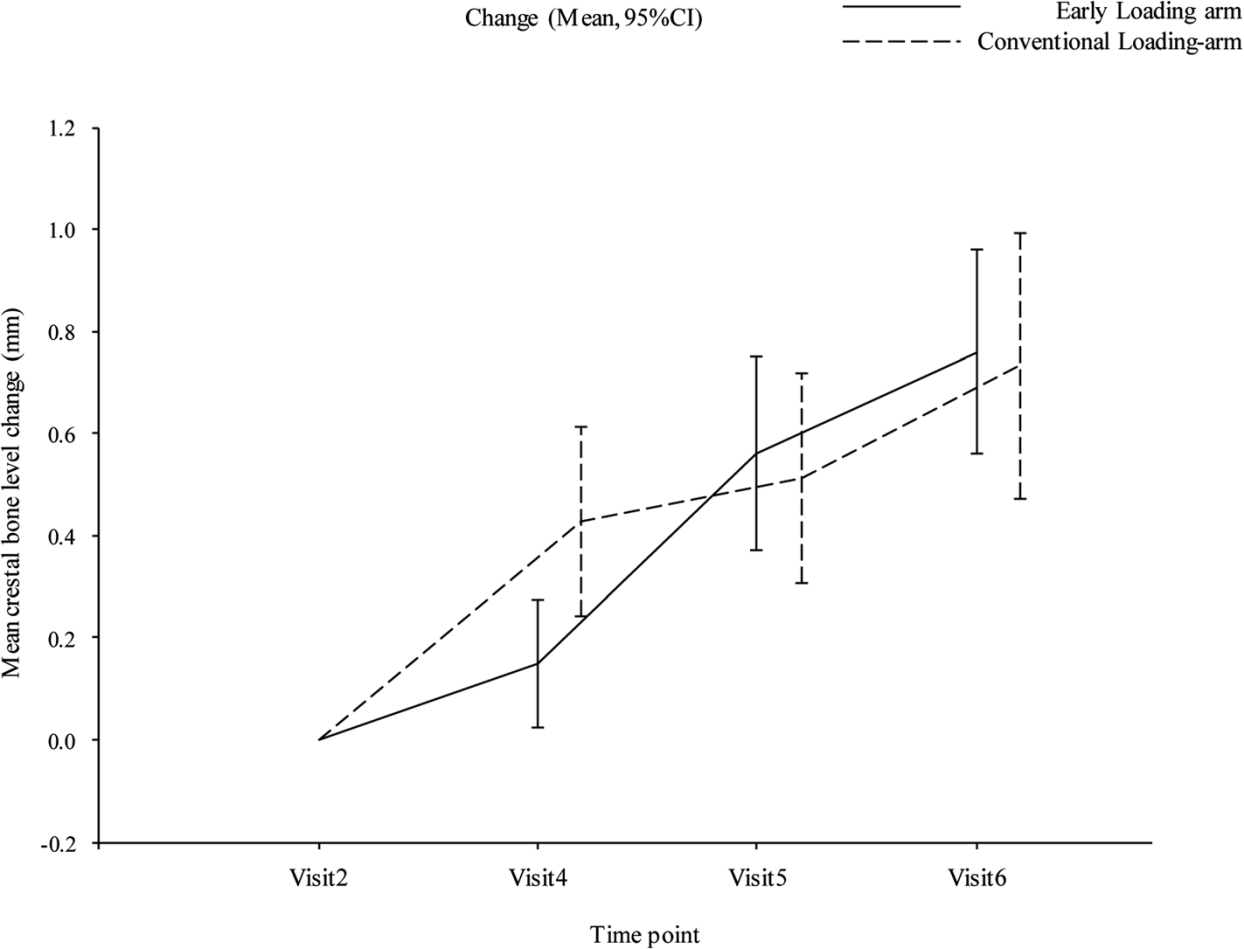
PPS mean crestal bone level change plotted against time (mean, 95 % CI)

The mean change in crestal bone level in the PPS 6 months after implant placement was 0.56 ± 0.58 and 0.51 ± 0.62 mm in the early and conventional loading arms, respectively, while at 12 months, it was 0.76 ± 0.60 and 0.73 ± 0.77 mm, respectively (Table 4 and Fig. 4). The inter-arm differences in the means between the early and conventional loading arms 6 and 12 months after implant placement were 0.048 (95 % CI −0.227–0.322) and 0.026 mm (95 % CI −0.293–0.346), respectively. Non-inferiority, defined as crestal bone level change between the treatment arms of ≤0.3 mm, was therefore confirmed. The mean change in crestal bone level in the FAS 6 months after implant placement was 0.56 ± 0.58 and 0.51 ± 0.62 mm in the early and conventional loading arms, respectively, while at 12 months, it was 0.78 ± 0.61 and 0.73 ± 0.77 mm, respectively. The inter-arm differences in the means between the treatment arms in FAS 6 and 12 months after implant placement were 0.048 (95 % CI −0.227–0.322) and 0.045 (95 % CI −0.271–0.362), respectively.

**Table 4:** Mean crestal bone level changes, in early loading and conventional loading arms (PPS), 6 months after implant placement

Treatment arm	Summary statistics	Baseline	6 months	Change from baseline to 6 months	12 months	Change from baseline to 12 months
Early loading-arm (*N* = 38)	No. of patients	38	38	38	38	38
	Mean (SD)	1.342 (0.600)	1.903 (0.603)	0.561^a^ (0.576)	2.102 (0.483)	0.760^b^ (0.603)
	Maximum	2.73	2.96	2.21	3.47	2.15
	Third quartile	1.73	2.22	0.88	2.34	1.14
	Median	1.25	2.015	0.55	2.05	0.80
	First quartile	0.98	1.52	0.17	1.77	0.37
	Minimum	0.29	0.48	−0.51	1.24	−0.39
Conventional loading arm (*N* = 37)	No. of patients	37	37	37	36	36
	Mean (SD)	1.355 (0.724)	1.868 (0.521)	0.513^b^ (0.617)	2.099 (0.558)	0.734^b^ (0.77)
	Maximum	3.01	3.81	1.82	3.74	2.54
	Third quartile	1.85	2.07	0.94	2.45	1.185
	Median	1.43	1.83	0.49	2.02	0.61
	First quartile	0.76	1.58	0.07	1.735	0.14
	Minimum	0.27	0.83	−0.71	1.04	−0.66

^a^Inter-arm difference (6 months): difference in mean 95 % confidence interval 0.048 (−0.227–0.322)

^b^Inter-arm difference (6 months): difference in mean 95 % confidence interval 0.026 (−0.293–0.346)

### Efficacy evaluations: implant survival and success rates

In the PPS and FAS, the implant survival rate was 100 % after 12 months. In the PPS, the implant success rate was 100 % at all time points in both the conventional and early loading arms; however, in the FAS, the success rate in the conventional loading arm was 100 % at all time points, while in the early loading arm, success was 100 % at suture removal at 12-month follow-up and 95 % at the final restoration time point (6 months). This was because two patients were excluded from FAS because the insertion torque did not reach 15 Ncm and time point for loading was postponed. Finally, the implant treatments were also successful in these patients, hence the 100 % success rate at follow-up. Survival was therefore “not evaluated” instead of “surviving,” and success was “failed” instead of “successful” at 6 months.

### Efficacy evaluations: patient satisfaction

Patients in both the PPS early and conventional loading arms at the 12-month follow-up generally rated their satisfaction in the indicators of prosthetic comfort, appearance, ability to taste, ability to chew, and general satisfaction as “satisfied” or “highly satisfied.” However, for the satisfaction parameter of “fitting” (i.e., time take to occlusal loading after surgery), all patients in the early loading arm rated their satisfaction as either highly satisfied or satisfied (38 of 38 patients), while in the conventional loading arm only 78.4 % of patients (29 of 37) gave the same rating (Table 5). The results for FAS were similar to those outlined above.

**Table 5:** Patient satisfaction at 6 and 12 months, number of patients (%)

	Indicator	Prosthetic comfort^a^		Appearance^b^		Ability to chew^c^		Ability to taste^d^		Fitting^e^		General satisfaction^f^	
Treatment arm	Timeline (months)	6	12	6	12	6	12	6	12	6	12	6	12
Early loading *N* = 38	Highly satisfied	12 (31.6)	11 (28.9)	14 (36.8)	14 (36.8)	10 (26.3)	12 (31.6)	11 (28.9)	16 (42.1)	11 (28.9)	9 (23.7)	14 (36.8)	14 (36.8)
	Satisfied	25 (65.8)	26 (68.4)	22 (57.9)	24 (63.2)	26 (68.4)	24 (63.2)	25 (65.8)	21 (55.3)	25 (65.8)	29 (76.3)	23 (60.5)	24 (63.2)
	No opinion	1 (2.6)	0 (0.0)	2 (5.3)	0 (0.0)	2 (5.3)	2 (5.3)	2 (5.3)	1 (2.6)	2 (5.3)	0 (0.0)	1 (2.6)	0 (0.0)
	Dissatisfied	0 (0.0)	1 (2.6)	0 (0.0)	0 (0.0)	0 (0.0)	0 (0.0)	0 (0.0)	0 (0.0)	0 (0.0)	0 (0.0)	0 (0.0)	0 (0.0)
	Highly dissatisfied	0 (0.0)	0 (0.0)	0 (0.0)	0 (0.0)	0 (0.0)	0 (0.0)	0 (0.0)	0 (0.0)	0 (0.0)	0 (0.0)	0 (0.0)	0 (0.0)
Conventional loading *N* = 37	Highly satisfied	14 (37.8)	13 (35.1)	14 (37.8)	12 (32.4)	11 (29.7)	14 (37.8)	17 (45.9)	18 (48.6)	12 (32.4)	8 (21.6)	19 (51.4)	17 (45.9)
	Satisfied	21 (56.8)	24 (64.9)	20 (54.1)	24 (64.9)	23 (62.2)	21 (56.8)	16 (43.2)	18 (48.6)	16 (43.2)	21 (56.8)	17 (45.9)	19 (51.4)
	No opinion	2 (5.4)	0 (0.0)	2 (5.4)	1 (2.7)	3 (8.1)	2 (5.4)	4 (10.8)	1 (2.7)	7 (18.9)	6 (16.2)	1 (2.7)	1 (2.7)
	Dissatisfied	0 (0.0)	0 (0.0)	1 (2.7)	0 (0.0)	0 (0.0)	0 (0.0)	0 (0.0)	0 (0.0)	2 (5.4)	1 (2.7)	0 (0.0)	0 (0.0)
	Highly dissatisfied	0 (0.0)	0 (0.0)	0 (0.0)	0 (0.0)	0 (0.0)	0 (0.0)	0 (0.0)	0 (0.0)	0 (0.0)	1 (2.7)	0 (0.0)	0 (0.0)

^a^Does the patient have any uncomfortable feeling about the placed implant?

^b^What does the patient think about the prosthesis appearance?

^c^What does the patient think about chewing?

^d^Does the patient have any uncomfortable feeling about taste?

^e^What does the patient think about the time taken until occlusal loading was started after implantation surgery?

^f^Is the patient generally satisfied with the treatment result?

## Discussion

This was a randomized, controlled, multicenter clinical trial to investigate whether the outcomes for chemically modified SLA implants in terms of change in crestal bone level from implant surgery to 6 months were non-inferior with early loading (25 ± 3 days) compared to conventional loading (13 ± 1 weeks). The difference in mean crestal bone level change between the early loading and conventional loading groups was 0.048 mm (95 % CI −0.227–0.322); non-inferiority of early loading was therefore confirmed within the parameters of the study. Therefore, null hypothesis was rejected.

Few randomized clinical trials are available showing the results of early loading with chemically modified SLA implants, and there are also relatively few prospective observational studies available [41, 42]. However, clinical studies have shown that successful osseointegration can be maintained and achieved for up to 3 years with these implants, with lower probing depth and clinical attachment level values compared to historical SLA controls [35, 43, 44]. Short implants with this surface have also shown high survival rates and good crestal bone levels after 2 years [37]. Early loading with SLA implants has been shown to be predictable, with excellent outcomes. Clinical data have shown that SLA implants can have very high success rates after 5 years following restoration after 6 weeks in type I to III bone and after 12 weeks in type IV bone, in both fully edentulous and partially edentulous patients and in both the mandible and maxilla [45–48], with stable crestal bone levels over 5 years [49]. Predictable early loading of SLA implants with maxillary full-arch prostheses [50] and mandibular overdentures [51] has also been observed.

The mean change in the bone level between baseline and 6 and 12 months of 0.561 and 0.760 mm, respectively, for early loading was similar to that found in other clinical studies of early loading with chemically modified SLA (SLActive®) implants. For example, a mean change in the bone level of 0.63 ± 0.95 mm from baseline to 12 months was observed with early loading of implants with the chemically modified SLA surface in the posterior maxilla and mandible in a large prospective multicenter study [36]; after 3 years, the mean change in crestal bone level was 0.88 ± 0.81 mm, indicating minimal further bone loss beyond 12 months [38]. An earlier study of early loading with implants with the SLA surface showed a mean bone loss of 0.52 ± 0.98 mm after 1 year, also in the posterior maxilla and mandible [52]. A three-arm study of early loading of SLA implants in the edentulous posterior maxilla and mandible and completely edentulous maxilla showed a mean marginal bone loss of 0.75 ± 1.3 mm after 1 year [53], while a study of SLA implants in the posterior mandible showed mean crestal bone loss values of 0.57 ± 0.49 and 0.72 ± 0.50 mm for early loading after 2 and 6 weeks, respectively, after 1 year [54].

The implant survival rate of 100 % after 12 months is also in line with the results from previous studies with chemically modified SLA implants in various situations, including 100 % survival in early loading of mandibular overdentures [55], 100 % survival in single-tooth applications in the anterior maxilla [56], 96.8 % with maxillary sinus floor augmentation [57], and 98 and 97 % with immediate and early loading in posterior jaws [36], respectively. Excellent implant survival rates with early loading have also been achieved over longer time periods; for example, with chemically modified implants loaded after 21 days, 100 % survival and success were observed over 3 years in the posterior mandible [35].

Implant survival rates in the current study also compare well with survival and success rates obtained for SLA implants over an equivalent period of time. For example, Al-Nawas and colleagues achieved implant survival rates of 96.9 and 96.4 % for SLA implants loaded after 4 and 12 weeks, respectively [58], while Fischer and Stenberg achieved 100 % survival with SLA implants supporting early-loaded maxillary full-arch prostheses [59]. It should be remembered, however, that the patients enrolled in the current study were treated by highly experienced implant surgeons and were subjected to strict inclusion and exclusion criteria, although high survival rates with these implants have also been found in daily dental practice [60, 61].

The study was performed because early loading protocols for dental implants in Japan are still relatively uncommon, even though early loading of dental implants has elsewhere been demonstrated to be a viable treatment option to restore esthetics and function to the patient in a timely manner [12, 15, 16, 45] and has shown advantages in terms of preservation of hard and soft tissues [14], as well as providing psychological benefits for the patient. Few clinical trials on early loading have been conducted in the Japanese population, and the authors speculate that this may indicate a more conservative approach to implant rehabilitation among Japanese dentists and implant surgeons. However, a recent prospective, multicenter, non-interventional analysis of Straumann bone level implants with the chemically modified SLA surface in daily dental practice indicated that conventional loading is still very much the norm in most countries. In this analysis, the authors found that of 1113 implants, 68.6 % were loaded with a conventional loading protocol, while early loading was used in only 12.4 % of cases [61]. Conventional loading is therefore still very much favored, despite evidence that there are no clinically important differences between the different loading protocols regarding implant or prosthesis failure [12, 62] or crestal bone loss [12, 15]. It has been noted, however, that not all clinicians can achieve optimum results with early loading and that high primary implant stability may be a requirement for a successful procedure [62].

Early loading of the implants showed a good safety profile, with a similar incidence in AEs between the early and conventional loading groups. The benefits to the patient for the early loading procedure were demonstrated by the patient satisfaction question “What does the patient think about the time taken until occlusal loading was started after implantation surgery?” All patients in the early loading group were “satisfied” (76.3 %) or “highly satisfied” (23.7 %), compared with 78.4 % of patients in the conventional loading group who were “satisfied” (56.8 %) or “highly satisfied” (21.6 %). Scores for the other categories of patient satisfaction were similar between the groups, corresponding to similar levels of patient satisfaction with early and conventional loading in other studies with single-tooth implant rehabilitation [63, 64].

The authors recognize that the study has certain limitations. For example, 6 months is a relatively short time for evaluation of a primary efficacy endpoint; generally, a minimum of 1 year is required for scientific validity in implant dentistry. Although the same evaluation as the primary efficacy endpoint (i.e., change in crestal bone level from baseline) was measured at 12 months, in retrospect, the change at 12 months should perhaps have been taken as a more clinically relevant primary efficacy endpoint. Increasingly, clinicians are calling for long-term evidence on dental implants and, as the time that implants have remained in situ in patients has increased over the years, more and more studies of 10, 15, and 20 years and over are being published and show high survival rates and low crestal bone loss [6, 65–71].

Since the softer bone in the maxilla may lead to a greater incidence of late implant failure [72], and therefore may require a longer loading protocol than the early loading protocol in this study [73], in retrospect, it may have been valid to evaluate the outcomes in the posterior maxilla and mandible separately. Similarly, because of the differences in ridge dimensions from premolar to molar sites in both jaws [74], a separate analysis of the crestal bone changes in these areas may have been applicable.

## Conclusions

In conclusion, this study demonstrated that early implant loading was non-inferior to conventional implant loading in terms of crestal bone level change in a Japanese patient population in short follow-up period and single tooth gaps in molar regions. High implant survival and patient satisfaction rates, and a good safety profile, were also achieved.
